# Commentary: Developing best-practice guidelines for the reporting of E-values

**DOI:** 10.1093/ije/dyaa094

**Published:** 2020-08-02

**Authors:** Tyler J VanderWeele, Maya B Mathur

**Affiliations:** 1 Departments of Epidemiology and Biostatistics, Harvard T.H. Chan School of Public Health, Boston, MA, USA; 2 Quantitative Sciences Unit, Stanford University, Stanford, CA, USA

We would like to thank Blum *et al*.^1^ for their interesting analysis of the current reporting practices around the use of the E-value to evaluate sensitivity to unmeasured confounding.^2^^,^^3^ As with nearly any quantitative tool, the E-value is potentially subject to misuse,^4^^,^^5^ examples of which are indeed documented in their paper. This arguably also points to the need for the development of best-practice guidelines for the reporting and interpretation of E-values. We will here offer some reflections on potential reporting guidelines.

## The need for sensitivity analysis

Blum *et al*.^1^ sampled a set of control papers from the same journals as those that reported E-values. Of these 69 papers, 52 (75.3%) apparently had no discussion whatsoever of unmeasured confounding. Unmeasured confounding is a major threat in most observational studies. That this threat is left both undiscussed and unquantified so frequently is troubling. Some form of sensitivity analysis or critical assessment of potential uncontrolled confounding is needed to address this problem. The E-value was developed as a particularly straightforward approach to do so,^3^^,^^4^^,^^6^ but there are of course other tools available.^7^^,^^8^ However, some approach should be employed. As noted in our paper^3^ and by Blum *et al*.^1^, the E-value is not context-free. The E-value needs to be evaluated in light of the measured confounders, the outcome, the exposure and the potentially known unmeasured confounders.^2–4^^,^^9^

## Identifying known unmeasured confounders

Whenever possible, it would be good to report specific variables that are thought to be potential unmeasured confounders. Blum *et al*.^1^ report that, of the 87 articles in their sample, 34 (39%) named specific variables that could be confounders and were unaccounted for. Such reporting should be improved. There are settings in which all known risk factors for an outcome are controlled for and, in such circumstances, it is not possible to specifically name a potential unmeasured confounder, but it is unlikely that this constitutes the remaining 61% of cases. Even in such settings wherein no specific unmeasured confounder can be named, it can be worthwhile calculating E-values or performing some other sensitivity analysis, as unknown unmeasured confounders can still be a threat. However, as a general principle, it would be good if all papers either stated that control was made for all known confounders or alternatively discussed which important unmeasured confounders might still have biased the analysis.

## Assessing confounding association magnitudes

Blum *et al*.^1^ rightly emphasize the need to interpret the magnitude of the required confounding associations. In our papers,^2–4^ we have not provided ‘cut-offs’ for what were large or small E-values. That will be relative to the outcome and exposure under consideration.^2–4^^,^^9^ It will also be relative to what measured confounders have been adjusted for.^2–4^^,^^9^ If adjustment has been made for numerous measured confounders related to the unmeasured variable, then the residual confounding associations are likely to be small. In contrast, if there are multiple unmeasured confounding variables, then it is possible for the residual confounding associations to be very large and, in such cases, not even a large E-value would provide much evidence for causation.^2–4^^,^^9^ However, if there are several (e.g., three or four) distinct important known unmeasured confounders, then a reasonable effect estimate likely cannot be obtained to begin with (these would not be the right data with which to attempt to address the research question). The E-value approach, and sensitivity analysis more generally, will be most helpful when there is a single known unmeasured confounder, or when adjustment has been made for all known measured confounders but, of course, with the possibility still of an unknown unmeasured confounder.

With a single known or unknown unmeasured confounder, it is still important to have some sense as to how large the confounding associations may be. Previous studies that have measured the variable in question (if there is a known unmeasured confounder) can be helpful in this regard, as would be the field-wide systematic umbrella reviews that Blum *et al*. support. One challenge with such approaches is that covariate associations with outcomes can vary with what other covariates have been adjusted for, and can also vary across populations. Another approach to trying to gain some sense as to what constitutes a small or large E-value in a given context would be to report associations of each of the measured covariates with the outcome. This could be done in an online supplementary table, with some comment given in the main text. In outcome-wide settings in which numerous outcomes,^10^^,^^11^ and thus numerous E-values, are assessed, a single table perhaps reporting the three largest covariate–outcome associations (properly inverted for protective associations) for each outcome could once again be helpful. In principle, a similar approach might also be used for the exposure, by providing the largest exposure–covariate relative risk associations across covariates. This would be straightforward for binary covariates but would require, for example, median dichotomization of the covariates, or some other approximate approach, for ordinal or continuous covariates. In any case, all of these practices may help inform what might be considered a large or small E-value in a given context and the extent to which unmeasured confounding may shift estimates.

## E-values for the confidence limits

We have previously emphasized the importance of reporting of E-values for the confidence interval.^3^^,^^9^ In Blum *et al*.’s study,^1^ the median E-value for the confidence interval for studies where confounding was deemed ‘unlikely to affect’ results (E-value = 1.49) was larger than that for studies in which confounding was deemed ‘likely to affect’ results (E-value = 1.28). That is at least encouraging, although no formal statistical test of the difference was given. Rather troubling, however, is that, according to the interquartile range reported by Blum *et al*.^1^ of studies that deemed unmeasured confounding ‘unlikely to affect’ the results, at least a quarter of the studies reported an E-value of 1 for the confidence interval. Presumably, many of these studies only reported the E-value for the estimate. Indeed, Blum *et al*.^1^ note that, of 87 articles, 33 (37.9%) reported E-values only for the estimate. Standard practice should be reporting E-values for the confidence interval as well.

## Requiring sensitivity analysis

Blum *et al*.^1^ note that there may be selective reporting of E-values, whereby E-values are more likely to be reported when the magnitude falls in line with the authors’ intended conclusion. Indeed, the E-values for the confidence interval in Blum *et al*.’s sample appear to be somewhat larger than those in a recent paper that attempted to calculate E-values from field-wide effect estimates regardless of whether or not E-values were reported by the original authors.^12^ This is exactly what one would expect if investigators decided to report E-values when they were large and omit sensitivity analysis for unmeasured confounding otherwise. Such selective reporting of E-values or sensitivity analysis seems likely to be pervasive until journal editors require some form of sensitivity analysis for unmeasured confounding in observational studies.^3^^,^^13^^,^^14^ As in our earlier paper, it would arguably be good practice if ‘in all observational studies intended to produce evidence for causality, the E-value be reported or some other sensitivity analysis be used’. There are plenty of other more extensive sensitivity analysis techniques;^1^^,^^7^^,^^8^^,^^15^ the E-value is just one particularly straightforward approach. Indeed, if the recent discussion around E-values^1–6^^,^^9^^,^^12–14^ got researchers to more frequently use various other more extensive sensitivity analysis approaches, we would likewise consider that a very successful outcome. However, good practice would be to require some formal assessment.

## Summary

The E-value is a relatively new simple approach to sensitivity analysis for unmeasured confounding. Guidelines for its use, reporting and interpretation are likely to evolve over time but in light of the above considerations, when the E-value is used, it would be good to ensure: (i) that this be reported for the confidence interval in addition to estimate; (ii) that authors provide some discussion as to what specific potential unmeasured confounders might be; and (iii) that authors compare the E-value with covariate–outcome associations from previous literature that may have had data on the confounder that was unmeasured in the present study and/or compare the E-value with covariate–outcome associations among the study's measured covariates. These recommendations are consistent with broader recommendations for the practice of bias analysis more generally.^15^ We have been endeavoring to carry out these reporting practices in our own work.^16^ Such reporting practices would arguably help prevent the misuse and misinterpretation of the E-value and would facilitate its proper use as an assessment of the sensitivity or robustness of results to potential unmeasured confounding.

## Funding

This research was support by NIH grant CA222147.

## Author contributions

T.J.V. drafted the manuscript and M.B.M. provided critical review and revision.

## Conflict of interest

None declared.
